# Past, Present and Future of Sensors in Food Production

**DOI:** 10.3390/foods3030491

**Published:** 2014-08-19

**Authors:** Catherine C. Adley

**Affiliations:** Microbiology Laboratory, Department of Chemical and Environmental Sciences, University of Limerick, Limerick, Ireland; E-Mail: Catherine.adley@ul.ie; Tel.: +353-61-202-646; Fax: +353-61-202-568.

**Keywords:** food, pathogens, biosensors

## Abstract

Microbial contamination management is a crucial task in the food industry. Undesirable microbial spoilage in a modern food processing plant poses a risk to consumers’ health, causing severe economic losses to the manufacturers and retailers, contributing to wastage of food and a concern to the world’s food supply. The main goal of the quality management is to reduce the time interval between the filling and the detection of a microorganism before release, from several days, to minutes or, at most, hours. This would allow the food company to stop the production, limiting the damage to just a part of the entire batch, with considerable savings in terms of product value, thereby avoiding the utilization of raw materials, packaging and strongly reducing food waste. Sensor systems offer major advantages over current systems as they are versatile and affordable but need to be integrated in the existing processing systems as a process analytical control (PAT) tool. The desire for good selectivity, low cost, portable and usable at working sites, sufficiently rapid to be used at-line or on-line, and no sample preparation devices are required. The application of biosensors in the food industry still has to compete with the standard analytical techniques in terms of cost, performance and reliability.

## 1. Introduction

The consumer is dependent on quality food manufacturing processes. Contaminating microorganisms may enter and reach the end-product through raw materials, air in the processing plant area, process surfaces, or factory personnel. Spoilage bacteria may also build up in high numbers in processing equipment and develop into biofilm. The sources of spoilage bacteria are numerous, however personnel and the environment being the most prevalent. 

Microbial management during the food processing operations is strategic for preventing contamination and for improving the product safety, quality and production hygiene. Built in mechanisms for in-process sampling points and frequency is necessary. Risk assessment tools like Hazard Analysis Critical Control Points (HACCP) can be used to detect areas of a process that are at risk of contamination [1], in addition, approaches such as Failure Modes and Effects Analysis (FMEA), can be implemented as outlined in a salmon processing company [2]. European Union (EU) member state companies must adhere to the rules laid out on Food Hygiene Legislation [3]. This legislation lays out rules on food hygiene through both general requirements and more specific rules, including the layout of premises, temperature control, HACCP, equipment, transport of food, waste, personal hygiene and training of food handling personnel. Specific hygiene rules for food of animal origin are also in the legislation [4]. These regulations are updated and changed on an ongoing basis.

Contamination screening during food processing operations would allow the food company to preventively stop the production, thus limiting the damage to just a part of the entire batch with considerable savings in terms of product value. Many contamination events are from biofilm formation and result from ineffective cleanings and disinfection processes [5]. The downstream processing of food cannot always prevent microorganism from entering the systems and many types of equipment cannot be sterilized, hence process management is vital [6]. The testing of food quality has in the past mainly dealt with the characterization of chemical contamination of the food product and testing has included physicochemical, biological and serological test techniques (*i.e*., chromatography, spectrophotometry, electrophoresis, titration and others). Chemicals are generally analyzed using gas chromatography (GC) or high pressure liquid chromatography (HPLC). These methods are carried out to separate the components of a complex sample and identify them through specific types of detectors. Common detectors used include, flame ionization (FID) and thermal conductivity (TCD) for GC; ultraviolet light (UV), fluorescence (FL) or mass spectrometry (MS) for HPLC.

Microbiological testing has been based on traditional “growth” based methods. These methods relied on nutrient media and have provided the basis for quantitative microbial assay for microbial safety and quality product release. The time required to get results using these techniques is long and forward processing decisions and confirming manufacturing processes are static, results that may take days are now deemed to be inadequate. 

Analytical methods to detect food borne pathogens are still evolving. There has been a surge in rapid microbial methods in the literature but in general they break down into three main categories: Qualitative methods (ATP bioluminescence, electrochemical measurements, micro-calorimetry); Quantitative methods (flow cytometery, direct epifluorescence technology) and identification methods (fatty acid analysis, ribotyping, polymerase chain reaction (PCR)). Newer emerging technologies include Raman spectroscopy, direct laser based detection, quantitative Real Time PCR and sensors and lab on chip (LOC) methods. Newer mass spectroscopy (MS) innovative methods such as matrix assisted laser desorption ionization time of flight (MALDI-TOF), surface enhanced laser desorption ionization time of flight (SELDI-TOF) and Fourier transfer infrared (FT-IR) mass spectroscopy (MS) methods have emerged. However, these MS methods rely on using isolated colonies as starting materials. Nucleic acid amplification methodologies such as PCR, ribotyping and gene sequencing burst on the commercial scene and have proved to have some sustainability. As patents for commercial system (electrochemical mostly) expire, new players are entering the market.

Applications for monitoring technologies range across the food industry. These monitoring technologies encompass, process control (the moisture content of the food; viscosity and texture); pH and conductivity (acidity and salt content); sugar content (glucose and sucrose are the main sugars monitored); food freshness including the detection of microbes (*Escherichia coli*, *Salmonella*, *etc.*) and the detection of microbial toxins (liquid and gas); ingredient freshness (milk, meat, *etc.*); frying oil (viscosity and chemical make-up) and food quality including taste (electronic nose).

## 2. Food Borne Pathogens

The European Food Safety Authority (EFSA) and the European Centre for Disease Prevention and Control (ECDC) analyzed the information submitted by 27 European Union Member States on the occurrence of zoonoses and food-borne outbreaks in 2011 [7]. The term zoonoses cover infections and diseases that are naturally transmissible either directly or indirectly, for example via contaminated foodstuffs, between animals and humans. 

Campylobacteriosis, with 220,209 human cases confirmed in 2011, was the most reported zoonosis in the EU with broiler meat being the most documented source of infection [7]. Salmonellosis cases have shown a decrease with a total of 95,548 confirmed cases in 2011, down from 101,037 confirmed cases in 2010 [7]. This reduction is attributed to successful *Salmonella* control programmes in poultry populations. The bulk of *Salmonella* that has been detected has come from meat and products thereof. Recent updated Directive EU 218/2014 [8] enhances the process hygiene criterion for *Salmonella* in pig carcases. Numbers of confirmed human case of listeriosis have decreased to 1476 [7]. *Listeria* was rarely detected above the legal safety limit for ready-to-eat foods. Nine thousand, four hundred and eighty-five confirmed cases of verotoxigenic *Escherichia coli* (VTEC) infection were described in 2011, representing an increase of 159.4% when compared with 2010 [7]. This was as a result of the large outbreak that happened, primarily in Germany, of Shiga toxin-producing *E. coli*/verotoxigenic *E. coli* (STEC/VTEC) that caused 54 deaths.

A total of 5648 food-borne outbreaks were reported in the European Union in 2011. These outbreaks resulted in 69,553 confirmed human cases, 7125 hospitalisations and 93 deaths [7]. The majority of the reported outbreaks were found to be caused by *Salmonella*, bacterial toxins, *Campylobacter* and viruses; however, the outbreak with most human cases was caused by STEC/VTEC and associated with sprouted seeds in Germany and France. The food sources most associated with these outbreaks were eggs and egg products, followed by mixed foods and fish and its products [7]. The full surveillance report for 2011 [7] including data in table format for each country can be obtained from the EFSA webpage. It must be remembered that the report relies on full compliance for reporting by the EU member states and some states are more diligent and established than others to date.

## 3. Biosensors

An analysis of the word “sensors” in the ISI Web of Science showed 433,020 hits for sensors from 1945–2014, however if one screens for food borne pathogens within this cohort only 47 articles are listed [9]. The first sensor developed, detected glucose using the enzyme glucose oxidase immobilized on a platinum electrode [10]. The first commercial glucose sensor was from the Yellow Springs Instrument (Model 23 YSI) and it reached the market in 1974. The instrument directly measured whole blood glucose levels from a 25 µL with a ±2% accuracy. The US Food and Drug Administration (FDA) have identified the YSI Model 23A and subsequent designs as the reference standard for measuring glucose [11]. Later antibodies in conjunction with optical transducers were developed for real time bioaffinity monitors. Blood glucose measurement still comprises about 85% of the world market for biosensors.

The biosensor market is highly competitive and is driven mainly by the medical and pharmaceutical sector. Market analysis in 2010, estimate that global revenues for biosensors will demonstrate robust growth and exceed $14 billion mark in 2016, with 47 different end user applications [12]. The bulk of the market in 2009 was for glucose sensors and toxicity testing, food borne pathogens including *E coli*, *Salmonella*, *Listeria*, is a small percentage of this market [12,13]. The growth in the market will be from security and biodefense, environmental monitoring, home diagnostics and process industry market sectors. Further developments of sensors are likely in the following areas: inherent accuracy, capability, intelligence, reliability, smaller sizes, power consumption, packaging, lower costs, and the elimination of lead. Despite the vast number of publications and reports, the field of biosensors comprises two broad categories (1) sophisticated, high throughput laboratory machines capable of rapid accurate measurement of complex biological interactions and components and (2) easy to use portable devices for use by non-specialists for in situ or home monitoring. Further developments are expected to be in the areas of Micro-Electro-Mechanical Systems (MEMS) and nanotechnologies. Sensors developed for industries such as the motor industry are been translated to human heart and motion monitoring. 

A key feature of the biosensor market is the large number of industrial alliances and licencing agreements. New approaches including molecular imprinting polymers (MIP) [14] as generic alternatives to antibodies, which allow selected functional monomers to self-assemble around a target analyte, is expanding sensor applications. The resulting MIP structures contains cavities which reflect both the shape and chemical functionality of the target species [15] with advances in reusable (up to 30 times) molecular templates developing [16,17]. During the last few years, mass-sensitive acoustic transducers, in particular the quartz crystal microbalance (QCM), have become very popular in combination with imprinted polymers [18]. There have also been recent serious discussions about harnessing the capabilities of smart phones as sensing tools [19]. 

Market challenges include, regulatory compliance, extended product lifecycles, reduced product development time, and product safety [12,13]. A significant number of reviews on sensors are available [20,21,22]; some are specific to food borne pathogens [23,24] and in specific application such as endotoxins [25], mycotoxins [26]; species specific reviews on *Campylobacter* spp. [27], *E. coli* non 0157 [28], recent trends in antibody sensors [29] and other reviews which deal with pesticides [30], milk [31], food processing [32]; nanomaterials [33,34]; conducting polymers [35] and molecular imprinted polymers [18].

## 4. Biosensor Component

**Figure 1:**
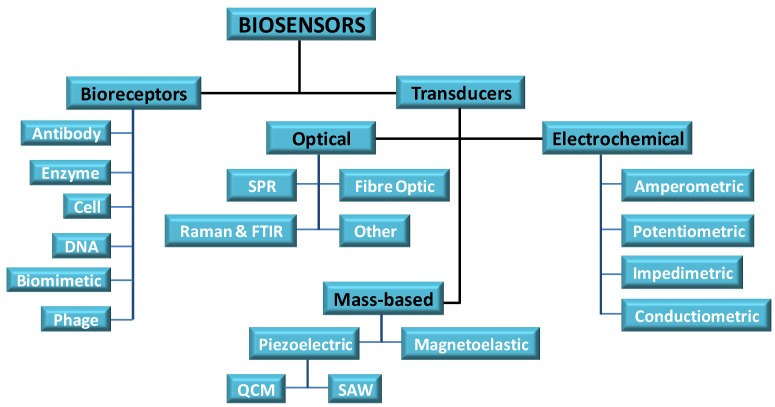
Components of a biosensor.

A biosensor is an analytical device that converts a biological response into a detectable measurable signal. A number of stages must be realised in developing a biosensor (Figure 1). *Transduction*, *signal generation* (increase of signal or reduction of noise); *fluidic design* (sample injection and drainage, concentration of sample, reduction of sample consumption, increase of analyte transport, reduction in detection time); *surface immobilization chemistry* (analyte capture efficiency, elimination of nonspecific binding); *detection format* (direct binding, sandwich type binding, competitive binding) and *data analysis* (extraction of information regarding analyte concentration, binding kinetics) [36]. Taking all of these considerations together a biosensor is made up of three components: the sensor material base has traditionally being made of metal, glass, polymer or even paper, onto which a bioreceptor is coupled. The bioreceptor (antibodies, enzymes, nucleic acid aptamers or single stranded DNA, cellular structures/cells, biomimetic and bacteriophage (phage) [24], is coupled in the sensor through a number of immobilizing techniques which can be physical or chemical. Chemical groups that are reactive can include functional groups such as carboxyl, –COOH; amine; –NH_2_; and hydroxyl, –OH. As environmental factors can affect biological materials making them very sensitive, they can easily lose their activity when forced to interact with the solid surface. The methodology for surface attachment of the probe is the most important step in fabrication of biosensors and requires a high level of control over the surface chemistry present. 

The trend in biosensors to date include, enzyme, antibody or antigen based biosensors; gene based sensors and whole cell sensor. Enzyme-based biosensors dominate the market and are mostly based on electrochemical transduction systems with glucose oxidase sensors dominating the market, the other focus are on chemical determinants (e.g., toxins, pesticides). However, many conjugated polymer based biosensors rely on indirect detection of the target analyte, usually a fluorescently labelled compound and this is especially true for biomolecular macromolecules such as proteins. Fluorescent sensors using boronic acid as a ligand, in a non-enzymatic approach for the detection of saccharides have found applications in microbial detection, as polysaccharides are a component of the bacterial cell membrane [37].

The third component which is vital is the transducing element. The bioreceptor should bring about a physio-chemical change that is measurable in close proximity to the transducer when it engages the target analyte. This change must produce a measurable signal that is proportionate to the concentration of the bioreceptor/target interaction. The signal can be measured by different techniques such as electrochemical, optical techniques, *etc.* (Figure 1). The sensor surface should be in an inactive or passive state when a measurement is not being conducted. For reusable sensors, after the measurement is completed, the target species is expelled by an external stimulus and the surface returns to its inactive form.

## 5. Sensor Materials

**Figure 2:**
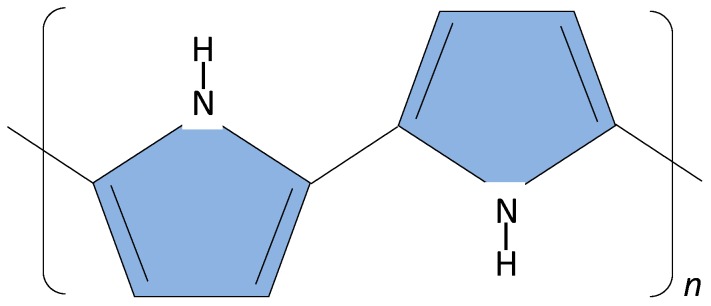
Structure of polypyrrole.

The sensor surface can be made of metal, polymer, glass or paper. Conducting polymers are polymer materials with metallic and semiconductor characteristics, a combination of properties not exhibited by any other known material. A key property of a conductive polymer is the presence of conjugated double bonds along the backbone of the polymer. In conjugation, the bonds between the carbon atoms are alternately single and double. The most common types of conjugated polymers are poly(acetylene)s, poly(pyrrole)s, poly(thiophene)s, poly(terthiophene)s, poly(aniline)s, poly(fluorine)s, poly(3-alkylthiophene)s, polytetrathiafulvalenes, polynapthalenes, poly(*p*-phenylene sulfide), poly(*p*-phenylenevinylene)s, poly(3,4 ethylenedioxythiophene), polyparaphenylene, polyazulene, polyparaphenylene sulfide, polycarbazole and polydiaminonaphthalene. They have found extensive use in the creation of electrochemical sensors such as potentiometric, amperometric and conductometric sensors [38]. Polyaniline followed by polypyrrole and polythiopene are the most used [39]. The structure of polypyrrole is shown in Figure 2.

In work carried by our group we developed a polymer nanocomposite sensors using polyethylene adipate (PEA) for a gas sensor for quantification of bacterial cultures [40]. In addition we used polypyrrole in the detection of *Bacillus cereus* [41], developing unique DNA primers which could differentiate between the *B. cereus* group spp., in spiked milk [42].

Metal-organic framework (MOF) materials have recently been explored as chemical sensors. MOF’s are extended crystalline structures where the metal cations or clusters of cations (“nodes”) are connected by multitopic organic “struts” or “linker” ions or molecules [43]. Their importance is in their tunability and structural diversity. Improving detection by coupling MOF’s with vibrational spectroscopy such as surface enhanced Raman scattering (SERS) has shown additional promise. MOF’s have been recently applied in the development of glucose sensors in a non-enzymatic approach [44] and for the detection of dipicolinic acid (pyridine-2,6-dicarboxylic acid) a unique compound in bacterial spores [45]. Paper based sensors emerged as an alternative surface for sensors. Paper is thin lightweight and flexible. The main constituents of paper are cellulose fibres. The paper absorbs and transports liquids by capillary force without additional mechanical assistance; however they still suffer from limitations, including accuracy and sensitivity [46]. Among the patterning methods employed for deposition of functional materials on paper substrates, the inkjet printing method was advanced due to its ability to deposit precise amount of materials rapidly and ability to perform computer-controlled printing on specific locations [47]. Using paper and species specific enzymes with a colorimetric reporting system has been outlined for select food borne pathogens (*L. monocytogenes*, *E. coli* 0157:H7 and *S.* Typhimurium) with a reduced enrichment time and a LOD of 10^4^ CFU/mL [48]. 

Nanomaterials show similar dimensions to biomolecules like proteins and DNA. The integration of nanomaterials with biomaterials has developed into a study called nanobiomaterials. Nanostructured biomaterials have been projected to be the next stage in development of many devices, including in sensor technology with unique capabilities for data collection, processing and recognition with minimal false positive counts. Carbon nanotubes (CNT’s) are conducting, act as electrodes, and generate electrochemiluminescence (ECL) in aqueous solutions. They can be derivatized with functional groups (carboxylic, carbonyl and hydroxyl) that allow immobilisation of biomolecules either through covalent or non-covalent bonding [49]. The variety and range of sensor materials can be seen from using double layer gold nanoparticles and chitosan to detect *Bacillus cereus* [50] in an electrochemical immunosensor approach with a detection limit of 10.0 CFU/mL, in pure culture. Colloidal gold is one of the most studied nanomaterial available for biosensors, albeit it is expensive for large scale applications. Multiplexing using a carbon screen printed array to detect *E. coli* 0157:H7 and *E. sakazakii* (*Chronobacter*) and multiwalled carbon nanotubes with horse radish peroxidase (HRP) gave a LOD of 3.27 × 10^3^ CFU/mL and 4.5 × 10^3^ CFU/mL respectively [51]. Quantum dot nanoparticles and anti-*Salmonella* polyclonal antibodies immobilised by streptavidin biotin binding achieved a detection limits of 4 × 10^3^ CFU/mL in food extracts, using a custom built fluorometer to detect the fluorescent light [34]. Oligonucleotides immobilised on nanopillar arrays of silicon was fabricated to target ssDNA and measuring the refractive index with an ellipsometer, as a new approach in a label free optical sensor [52]. A selection of immunosensors has been reported for food borne pathogens including *E. coli* 0157:H7 using modified graphene paper and gold nanoparticles with antibody and biotin streptavidin system with a detection limit of 1.5 × 10^2^ CFU/mL [53]. A screen printed carbon electrode/carbon nanotube was developed to detect *E. sakazakii* in the range of 10^3^–10^9^ CFU/mL and a detection limit of 7.7 × 10^–1^ CFU/mL with long term storage capabilities [54]. However limitation due to *E. sakazakii* growth in milk powder after addition of water and delayed use was highlighted as a limitation. *Cronobacter* is now the officially recognised bacterial genus name for *Enterobacter*. A stable label-free electrochemical impedance immunosensor for the detection of *Salmonella* Typhimurium in milk was developed by immobilising anti-*Salmonella* antibodies onto gold nanoparticles and poly(amidoamine)-multiwalled carbon nanotubes-chitosan nanocomposite film modified glassy carbon electrode. A detection limit of 5.0 × 10^2^ CFU/mL was reported [55]. The application of a quartz crystal microbalance (QCM) instrument with a microfluidic system for the rapid and real time detection of *Salmonella* Typhimurim using immobilised anti-*Salmonella* antibody and gold-nanoparticles gave a sensitivity with a limit of detection (LOD) 10–20 CFU/mL compared to direct and sandwich assay (1.83 × 10^2^ CFU/mL and 1.01 × 10^2^ CFU/mL, respectively) [56]. Reviews on nanomaterials and biosensors as diagnostic tools and in food applications are available [33,57,58].

Nobel metals (e.g., gold, silver platinum, *etc.*) nanoparticles have been a focus. Numerous techniques to synthesis these nanoparticles and to control their properties (their size, shape and homogeneity) have been demonstrated. These techniques include both chemical methods such as chemical reduction, photochemical reduction, co-precipitation and hydrolysis, and physical methods such as laser ablation, grinding and vapor deposition [21]. Examples of food borne pathogen Nano Metal Particle (NMP) based sensors including electrical/electrochemical with gold NP to detect *E. coli* 0157:H7 in food samples at a LOD of 5.3 × 10^2^ CFU/mL [59] and *Salmonella* in pork samples with a detection limit of 1.0 × 10^2^ CFU/mL [60]. 

## 6. Sensor Designs

The technique used for the physical or chemical fixation of bioreceptor which can be cells, organelles, enzymes, or other proteins (e.g., monoclonal antibodies) onto a solid support, or into a solid matrix or retained by a membrane, is used in order to increase their stability. Methods used can be physical retention or chemical binding.

*Adsorption* is a physical method of immobilization. Many substances can adsorb enzymes and other biological materials on their surfaces for example alumina, charcoal, clay, cellulose, kaolin, silica gel, glass, collagen, carbon pellets and advanced material such as carbon nanotubes (CNTs). A simple procedure is when microbial cells are immobilized by simple absorption by placing the cells on a porous cellulose membrane. Generating pastes such as when enzymes or tissue are mixed with graphite powder and liquid paraffin.

*Entrapment*, physical method of immobilization: Entrapment means physical enclosure of biomolecule in a small space. Inert membranes have been used to provide close contact between the biomaterial and transducer. Types of membranes used include cellulose acetate (dialysis membrane); polycarbonate (Nucleopore), synthetic non-permselective material; Collagen, a natural protein; PTFE: polytetrafluoroethylene (trade name Teflon) and is a synthetic polymer selectively permeable to gases. Nafion, (a Dupont material), which is biocompatible and shown to be stable in cell culture and the human body. Polymeric gels can be used and prepared in a solution containing the biomaterial. Chemical polymers such as calcium alginate, carrageenan, polyacrylamide, and sol-gel (Sol-gel, is a glassy silica produced by polymerization of silicate monomers). 

*Bonding and cross linking*: a number of bonding mechanisms have been used including covalent bonding. A covalent bond exists between two atoms if they share electrons between them. The Biotin-Avidin bond is one of the strongest known non-covalent bonds. Avidin is a terameric protein that forms a highly specific binding site for Biotin. Sulphur compounds are known for their reactivity to metals and this absorb readily to the noble metals. Thiolised DNA can be attached to gold via different methods.

*Transducing element*: the transducing element must produce a measurable signal that is proportionate to the concentration of the analyte/bioreceptor. Transducers can be divided into optical, electrochemical and mass based (Figure 1). 

*Optical transducers* can be subdivided into light absorption, fluorescence/phosphorescence, reflectance, refractive index, bio/chemiluminiscence.

In reflectance three widely used methods are Surface Plasmon resonance (SPR), total internal reflection fluorescence (TIFR) and attenuated total reflectance (ATR). SPR has found some commercial instruments being developed by Biacore [61] for vitamin and antibiotic analysis of food. Using a polyclonal antibody against *L. monocytogenes* and a subtractive inhibition assay carried out with a BIAcore 3000 biosensor with a sensitivity of 1 × 10^5^ cells/mL comparable to ELISA tests has been reported [62]. Biosensing Instruments Ltd. [63] has developed an endotoxin detector also using SPR. Using a custom built SPR sensor based on ATR method and glass chips coated in gold and with streptavidin for biotinylated antibody binding for selected species (*E. coli* 0157:H7; *S. choleraesusi* serotype Typhimurium, *L. monocytogenes* and *C. jejuni*) provided limits of detection ranging from 3.4 × 10^3^ to 1.2 × 10^5^ CFU/mL. Both single and mixtures of the four species gave comparable results [64]. 

*Fiber optic biosensors* [65] and their application in food quality and safety [66] have been reviewed. Significant results in food matrixes to detect *Salmonella*, *E. coli* and *Listeria* was obtained, using streptavidin coated optical waveguides immobilized with biotinylated polyclonal antibodies in a multiplex reaction. The limit of detection for the sensor was ~10^3^ CFU/mL after 2 h for all pathogens [67]. However enrichment for 18 h was an initial step. 

*Electrochemical transduction* methods can be subdivided based on the measured parameter: amperometric (current), potentiometric (potential), impedimetric (impedance) and conductometric. The amperometric sensors have a superior sensitivity and better linear range than potentiometric devices and the most successful commercially. Most work has been done on amperometric and potentiometric biosensors with little work being devoted to conductometric biosensors [68]. Modern electrochemical techniques have low detection limits (10^−7^–10^−9^ M or 30 ppb) for gaseous compounds [69]. A range of detector components (antibody, DNA) have been used in the detection of *Campylobacter* spp. using both ampermometric and impedimetric transducers [27]. Electrochemical enzyme-based biosensors have dominated the market in the food sector including newer amperometric nanoparticles glucose sensors, based on hydrogel heterostructures with a response time of 3 s and sensitivity as high as 96.1 µA·mM^−1^·cm^−2^ [70].

*Mass based transducers*: mass sensitive biosensors are suitable for very sensitive detection, in which the transduction is based on detecting a small changes in mass. The two main types of mass based sensors are (1) bulk wave (BW) or quartz crystal microbalance (QCM) and (2) surface acoustic wave (SAW).

However, the detection of foodborne pathogens based on piezoelectric sensors are not versatile. A quartz crystal microbalance (QCM) immunosensor in the direct detection of *S.* Typhimurium in a chicken meat sample was demonstrated [71] which showed that the resonant frequency and motional resistance were proportional to the cell concentration in the range of 10^5^–10^8^ and 10^6^–10^8^ cells/mL, respectively. The detection limit was lowered to 10^2^ cells/mL by using anti-*Salmonella*-magnetic beads. A QCM is a real mass sensor belonging to a wider class of inertial mass sensors [72].

*Acoustic wave sensors* (AWS) monitor the change in oscillation frequency when the device responds to the input stimulus. The global AWS device market is expected to reach €1.8 billion by 2016 [73]. AWS can be subdivided into: (1) bulk acoustic wave resonators (BAW); (2) Flexural-plate-wav-resonators (FPW); (3) Surface acoustic wave resonators (SAW); and (4) shear-horizontal acoustic plate mode resonators (SAW). A review of SAW for the detection of pathogens is available [74]. An interesting SAW application is its use in an intelligent food packaging humidity monitoring system, consisted of a ZnO surface acoustic wave sensor directly built on the protein zein (a prolamine protein found in maize (corn)), measuring humidity for food freshness/protection [75]. Bulk acoustic wave have been used to detect proteins and DNA. Some applications in food to detect *E. coli* 0157:H7, *Salmonella* and *Listeria* have been summarised [76]. 

The overall features of a good sensor includes*: Selectivity*: the biosensor must be highly selective for the target analyte and have little or no cross reactivity with moieties that have a chemical structure similar to that of the target analyte. *Sensitivity*: the biosensor should be able to measure in the range of interest for a given target analyte with little in the way of additional steps such as pre cleaning and pre concentration of the samples. *Linearity of response*: the linear response range of the system should cover the same concentration range over which the target analyse is to be measured. *Reproducibility of signal response*: when samples having same concentrations are analyzed several times, they should give same response. *Quick response time and recovery time*: the time it takes for the biosensor to respond to the selected analyte should be quick enough so that real time monitoring can take place in an efficient manner. The recovery time of the sensor should be as small possible for reusability of the biosensor system. *Stability and operating life*: as such most of the biological compounds are unstable in different biochemical and environmental conditions [32].

## 7. Microbial Sensing

In order to detect microorganism in a liquid or solid sample multiple approaches have been undertaken. For the extensive amount of research generated there is limited commercial output. Approaches taken have been diverse from whole cell to cellular components.

### Microbial Whole Cell Biosensors

Sensors to detect whole cell bacteria have been slow to come to market, as microbial cells are complex and a sensor prefers a simple matrix in order to work efficiently. Microbial cells because of their low cost, long lifetime and wide range of suitable pH and temperature, have been explored. Some of the basic limitations of microbial biosensors as compared to enzyme sensors have been their long response time, low sensitivity and detection limits. Their slow response has been attributed to diffusional problems associated with the cell membranes. Systems reported using whole cell as sensors for ethanol in the food fermentation industry has commercial interest and multiple approaches have been taken and reviewed [77]. Using *Acetobacter aceti* and its respiratory membrane bound enzyme Alcohol dehydrogenase catalytic activity for ethanol measurement was an initial approach [78]. In many cases whole cell microorganisms have been used to detect chemical components such as environmental pesticides. Genetic engineered *Pseudomonas putida* JS444 was constructed to display organophosphorus hydrolase (OPH) activity on a dissolved oxygen electrode to detect synthetic organophosphate compounds (OP). In optimal condition it measured as low as 55 ppb for paraoxon, a potent acetylcholinesterase-inhibiting insecticides, without interference from other common pesticides [79].

Using genetically engineered microorganisms and enzymes is now the norm, including fusion proteins for tailoring sensors for specific purposes. In a new configuration for Biological Oxygen Demand (BOD) used to detect pollution problems, a chronamperometric response system, employed a double mediator system coupled with ferricyanide and a lipohhilic mediatator mendaione (synthetic compound) and *Saccharomyces cerevisiae* [80]. *P. syringae* was used as the biocatalyst to also measure BOD in water samples with a response time of 3–5 min, the biocatalyse was placed between cellulose and Teflon membranes [81]. A comprehensive list of electrochemical, conductometric, potentiometric whole cell microbial biosensors targeting a range of chemicals has been reviewed [82]. Commercialisation of whole cell biosensors has proved to be slow due to problems fabricating the whole cell to the appropriate surface and the stability of the microorganism. In the food processing industry applications of microbial whole cell biosensors in pathogen detection have not been embraced.

## 8. Nucleic Acid Sensors

Nucleic acid sensors have been the focus of much research. Several gene sensing detection methods for food borne pathogens have been developed with optical, electrochemical, mass sensitive and microgravimetioc techniques [24,83] and with multiplex PCR approached [84,85].

In the nucleic acid sensor, a DNA or RNA target is detected through the hybridization reaction between DNA or RNA and ssDNA sensing element. Examples of early DNA-based biosensor for *E. coli*, using PCR and piezoelectric quartz crystals was demonstrated to detect 23 cells per 100 mL water samples with application in public beach water quality regulations [86]. Other reports included using embedded *E. coli* DNA-uidA gene in polypyrrole [87] and in real time using a quart crystal microbalance using the eaeA gene (104bp) of *E. coli* 0157:H7 [88]*. Salmonella* spp. were the target using DNA streptavidin modified magnetic beads and electrochemical detection [89]. *L. monocytogenes* was detected using a magneto electrochemical luminescence PCR detection platform which gave a detection limit of 500 fb/µL genome DNA in 1 h [90]. The detection of *E. coli*, *Bacillus subtilis*, *B. atrophaeus* and *L. innocua* in meat juices demonstrated a detection limit of 500 CFU/*E. coli* in one working day [91] using esterase and an amplification based DNA array sensor. To enable large scale screening procedures, new multiplex analytical formats are being developed, and these allow the detection and/or identification of more than one pathogen in a single analytical run, thus cutting assay times and costs [92].

Microfluidic strategies coupled with electrochemical transducers have produced miniaturised devices. The lab-on-chip includes electrodes, hybridisation, washing and response. Label free detection using synthesised target DNA and real DNA samples from *S. choleraesusi* in dairy food was measured in real time [93]. The ability for microfluidic and multiplexing was demonstrated in an integrated system using gold nanoparticle labels for detection of *E. coli* and *B. subtilis* [94]. 

Real time detections is still a goal and coupled with PCR showed early developments [95]. A microchip with integrated modules for performing cell lysis, PCR, and quantitative analysis of DNA amplicons in a single step has been described for a lab-on-chip detection of *E. coli* O157:H7 and *Bacillus subtilis* [96]. This system however, demonstrated the classic shortcoming of temperature control in the PCR reaction. The application of loop meditated isothermal amplification (LAMP), has been demonstrated for *E. coli* and *S. aureus* using target genes amplified with LAMP using ruthenium hexamine as the intercalating electrochemical indicator [97]. 

The development of aptasensors has shown increased promise. Aptamers are DNA or RNA molecules that are selected from random pools and engineered through repeated rounds of *in vitro* selection based on their ability to bind other molecules; they can bind nucleic acid, proteins, small organic compounds, and even entire organisms. There are two main classes of aptamers—nucleic (DNA and RNA) aptamers and peptide aptamers. DNA and RNA aptamers typically consist of between 20 and 80 nucleotides. Aptamers have many advantages compared to antibodies as they can be produced easily and inexpensively. They are simple to modify chemically, label with different reporter molecules, to integrate into different analytical methods and can be coupled to different transduction systems [98]. Applications in food safety control have been reviewed [99], and in real food situations, e.g., *E. coli* using a potentiometric aptamer based biosensor with detection of 6 CFU/mL in milk and 25 CFU/mL in apple juice [100]. *Vibrio cholera* was detected at 0.85 ng/µL genomic DNA; DNAzyme aptamers for *Salmonella paratyphi* using nanotubes and fluorescence [101]. The design of the aptamers was carried out using SELEX (Systematic Evolution of Ligands by Exponential Enrichment). The engineering of aptamers using SELEX has caused recent excitement in the field of sensors since their discovery [98], and their applications have been explored in designing biomarkers, to treat cancer and in specific pathogen detection [102]. Gene-sensing methods gave initially very high hopes for rapid on line systems. Limitations include extraction of the DNA, dead cell detection even with the use of RNA to determine viability, the complex matrix of food, all provided ample false negatives results.

## 9. Sensors Using Bacteriophage 

Since their discovery by Twort and d'H´erelle, bacteriophages have not been universally exploited as control agents of disease. Although used in the former Soviet Union extensively, they did not translate into viable infection control options until recently. Bacteriophages are specific for certain bacteria and using this selectivity, phage typing has been extensively developed to differentiate between diverse strains of particular species of bacteria. Phage typing exploits their ability to specifically recognize molecules on the surface of the bacteria, to infect the cells and ultimately lyse their host. Phage as a detection system has come into the limelight comprehensively reviewed by Tawil *et al.* [103]; Schmelcher and Loessner [104]. 

## 10. Companies Developing and Producing Biosensors

The commercialization of biosensors lies in glaring contrast to the promise that is shown in the research literature. The global bio chip market is expected to reach US$11.4 billion by 2018 with a compound annual growth rate (CAGR) of 18.6% during 2012–2018. Biochip instruments are expected to exert the highest support to the industry with a CAGR of 20%. The microarray segment accounts for nearly 70% of the industry value [12,13]. 

However, rapid, lab on chip hand held systems are not forthcoming. Some systems are available including Nanosphere’s VeriGene Enteric Pathogens (EP)—a single use self-contained microfluidic cassette [105]. 3M has developed a number of systems for pathogen detection including *Salmonella* which received the Association of Analytical Communities (AOAC) Official Methods of Analysis Validation and an equivalent system for *Listeria.* 3M uses isothermal amplification of nucleic acids sequences with bioluminescence to detect the amplification. In addition there is the 3M™ Microbial Luminescence System (MLS) to detect the presence of microbial ATP in ultra-high treated (UHT) and extended shelf life (ESL) dairy end products [106].

Neogen [107] have a number of commercial food safety systems, the ANSR to detect *Salmonella* and *Listeria* uses isothermal amplification technology. The Reveal^®^ test system is an immunoassay with chromatography but requires enrichment. The NeoSeek™, targets seven STEC/*E.coli* strains, using enrichment with next day results. A mass spectrometry-based multiplexing system is the technology used. Their GeneQuence**^®^** detection assays utilize DNA hybridisation technology in a microwell format to detect *Salmonella*, *Listeria*, or *Listeria monocytogenes* and can run up to 372 samples at a time fully automated [107]. Serosep [108] have an EntericBio human stool samples to detect food borne pathogen which can be applied to food matrices. VereFoodborne™ is a nucleic acid-based, device, combines multiplex PCR and microarray hybridization to detect, differentiate and identify 15 of the major food pathogens [109], but has not achieved diagnostic validation. PDS Biophage Pharma [110] has advertised two systems PDS^®^ biosensor for total bacterial detection and bacTrapping which has phage on paramagnetic beads with magnetic separation. DetScan from Elice [111] is an electrochemical based sensor. Stratophase Ltd. (UK) Ranger™ Probe is an optical structure around a silicon chip that gives real-time, in-line bioprocess monitoring and fermentation control in food industries [112]. 

There are a number of prominent real-time PCR cycler manufactures which designed instruments for research with low capacities and others for high-throughput applications, Most employs fluorescent probes for detection with Quantitative PCR, multiplexing, HRM (high resolution melting), these include Roche, Agilient, Biacore, BioRad, Life Technologies-Applied Biosystems, A comprehensive listing of real time PCR instruments is available [113].

## 11. Conclusions

In determining the microbial control parameters in food, the spatial heterogeneity of the food matrix is not always taken into account. Microbial behaviour can be influenced by variables such as porosity, viscoelastic properties and the physicochemical attributes of foods, such as pH, water activity and the ability of nutrients and/or metabolites to diffuse. The microorganisms themselves can be influenced by the spatial and temporal heterogeneity of bacteria, the variability in the physiological stage in the cells, and the succession of the microbial community in time will all affect the sensors ability to detect. In addition, the stability and longevity of the sensing biomolecules under conditions in the field also need to be considered, e.g., is the sensor affected by temperature ranges, the presence of other chemicals and particulates? Simplified sample preparation procedures and separation techniques to selectively fractionate bacteria is also a limiting factor in sensor technologies.

Food inspecting agencies worldwide have a zero tolerance policy for the serious food borne pathogen organisms (*Salmonella*, *E. coli* 0157, *etc.*) presence in food. This zero tolerance must be the target for any new biosensor in its design and development to incorporate an inclusivity and exclusivity of detection in the systems. Sensor technology development has favored home diagnostics: point of care testing in healthcare; research laboratories; security and biodefense. The food industry has not embraced rapid method applications in food production and processing. Cost, performance and reliability have still to be addressed.

## References

[1] (1998). Food Quality and Safety Systems—A Training Manual on Food Hygiene and the Hazard Analysis and Critical Control Point (HACCP) System. http://www.fao.org/docrep/W8088E/W8088E00.htm.

[2] Arvanitoyannis I.S., Varzakas T.H. (2008). Application of ISO 22000 and failure mode and effect analysis (FEMA) for industrial processing of salmon: A case study. Crit. Rev. Food Sci. Nutr..

[3] (2004). Regulation (EC) No 852/2004 of the European Parliament and of the Council of 29 April 2004 on the Hygiene of Foodstuffs. Off. J. Eur. Union.

[4] (2004). Regulation (EC) No 853/2004 of the European Parliament and of the Council of 29 April 2004 Laying Down Specific Hygiene Rules for Food of Animal Origin. Off. J. Eur. Union.

[5] Chmielewski R.A.N., Frank J.F. (2006). Biofilm formation and control in food processing facilities. Compr. Rev. Food Sci. Food Saf..

[6] Cappitelli F., Polo A., Villa F. (2014). Biofilm formation in food processing environments is still poorly understood and controlled. Food Eng. Rev..

[7] (2011). The European Union Summary Report on Trends and Sources of Zoonoses, Zoonotic Agents and Food-Borne Outbreaks in 2011. EFSA J..

[8] (2014). Commission Regulation (EU) No 218/2014 of 7 March 2014 Amending Annexes to Regulations (EC) No 853/2004 and (EC) No 854/2004 of the European Parliament and of the Council and Commission Regulation (EC) No 2074/2005. Off. J. Eur. Union.

[9] Web of Science™. www.webofknowledge.com.

[10] Clark L.C., Lyons C. (1962). Electrode systems for continuous monitoring in cardiovascular surgery. Ann. N. Y. Acad. Sci..

[11] Chemical Heritage Foundation. http://www.chemheritage.org/discover/collections/collection-items/scientific-instruments/ysi-blood-glucose-analyzer-model-23a.aspx.

[12] Thusu R. Strong Growth Predicted for Biosensor Market. http://www.sensorsmag.com/specialty-markets/medical/strong-growth-predicted-biosensors-market-7640.

[13] Thusu R. Sensors Facilitating Health Monitoring. http://www.sensorsmag.com/specialty-markets/medical/sensors-facilitate-health-monitoring-8365.

[14] Turiel E., Martin-Esteban A. (2010). Molecularly imprinted polymers for sample preparation: A review. Anal. Chim. Acta.

[15] Haupt K., Mosbach K. (2000). Molecularly imprinted polymers and their use in biomimetic sensors. Chem. Rev..

[16] Poma A., Turner A.P., Piletsky S.A. (2010). Advances in the manufacture of MIP nanoparticles. Trends Biotechnol..

[17] Poma A., Guerreiro A., Whitcombe M.J., Piletska E.V., Turner A.P.F., Piletsky S.A. (2013). Solid-phase synthesis of molecularly imprinted polymer nanoparticles with a reusable template—“Plastic antibodies”. Adv. Funct. Mater..

[18] Vasapollo G., del Sole R., Mergola L., Lazzoi M.R., Scardino A., Scorrano S., Mele G. (2011). Molecularly imprinted polymers: Present and future prospective. Int. J. Mol. Sci..

[19] Ozcan A. (2014). Mobile phones democratize and cultivate next-generation imaging, diagnostics and measurement tools. Lab Chip.

[20] Turner A.P. (2013). Biosensors: Sense and sensibility. Chem. Soc. Rev..

[21] Doria G., Conde J., Veigas B., Giestas L., Almeida C., Assuncao M., Rosa J., Baptista P.V. (2012). Noble metal nanoparticles for biosensing applications. Sensors.

[22] Gehring A.G., Tu S.I. (2011). High-throughput biosensors for multiplexed food-borne pathogen detection. Annu. Rev. Anal. Chem. (Palo Alto Calif.).

[23] Arora P., Sindhu A., Dilbaghi N., Chaudhury A. (2011). Biosensors as innovative tools for the detection of food borne pathogens. Biosens. Bioelectron..

[24] Velusamy V., Arshak K., Korostynska O., Oliwa K., Adley C. (2010). An overview of foodborne pathogen detection: In the perspective of biosensors. Biotechnol. Adv..

[25] Das A.P., Kumar P.S., Swain S. (2014). Recent advances in biosensor based endotoxin detection. Biosens. Bioelectron..

[26] Vidal J.C., Bonel L., Ezquerra A., Hernandez S., Bertolin J.R., Cubel C., Castillo J.R. (2013). Electrochemical affinity biosensors for detection of mycotoxins: A review. Biosens. Bioelectron..

[27] Yang X., Kirsch J., Simonian A. (2013). *Campylobacter* spp. detection in the 21st century: A review of the recent achievements in biosensor development. J. Microbiol. Methods.

[28] Wang F., Yang Q., Kase J.A., Meng J., Clotilde L.M., Lin A., Ge B. (2013). Current trends in detecting non-O157 Shiga toxin-producing *Escherichia coli* in food. Foodborne Pathog. Dis..

[29] Holford T.R., Davis F., Higson S.P. (2012). Recent trends in antibody based sensors. Biosens. Bioelectron..

[30] Liu S., Zheng Z., Li X. (2013). Advances in pesticide biosensors: Current status, challenges, and future perspectives. Anal. Bioanal. Chem..

[31] Mortari A., Lorenzelli L. (2014). Recent sensing technologies for pathogen detection in milk: A review. Biosens. Bioelectron..

[32] Thakur M.S., Ragavan K.V. (2013). Biosensors in food processing. J. Food Sci. Technol..

[33] Ezzati Nazhad Dolatabadi J., de la Guardia M. (2014). Nanomaterial-based electrochemical immunosensors as advanced diagnostic tools. Anal. Methods.

[34] Kim G., Park S.B., Moon J.-H., Lee S. (2013). Detection of pathogenic *Salmonella* with nanobiosensors. Anal. Methods.

[35] Arshak K., Velusamy V., Korostynska O., Oliwa-Stasiak K., Adley C. (2009). Conducting polymers and their applications to biosensors: Emphasizing on foodborne pathogen detection. Sensors J. IEEE.

[36] Fan X., White I.M., Shopova S.I., Zhu H., Suter J.D., Sun Y. (2008). Sensitive optical biosensors for unlabeled targets: A review. Anal. Chim. Acta.

[37] Amin R., Elfeky S.A. (2013). Fluorescent sensor for bacterial recognition. Spectrochim. Acta Mol. Biomol. Spectros..

[38] Faridbod F., Norouzi P., Dinarvand R., Ganjali M.R. (2008). Developments in the field of conducting and non-conducting polymer based potentiometric membrane sensors for ions over the past decade. Sensors.

[39] Inzelt G. (2011). Rise and rise of conducting polymers. J. Solid State Electrochem..

[40] Arshak K., Adley C., Moore E., Cunniffe C., Campion M., Harris J. (2007). Characterisation of polymer nanocomposite sensors for quantification of bacterial cultures. Sens. Actuators B Chem..

[41] Velusamy V., Arshak K., Korostynska O., Oliwa K., Adley C., Kim M.S., Tu S.-I., Chao K. (2009). Conducting polymer based DNA biosensor for the detection of the *Bacillus cereus* group species. SPIE7315, Sensing for Agriculture and Food Quality and Safety.

[42] Oliwa-Stasiak K., Kolaj-Robin O., Adley C.C. (2011). Development of Real-Time PCR assays for detection and quantification of *Bacillus cereus* group species: Differentiation of *B. weihenstephanensis* and rhizoid *B. pseudomycoides* isolates from milk. Appl. Environ. Microbiol..

[43] Kreno L.E., Leong K., Farha O.K., Allendorf M., van Duyne R.P., Hupp J.T. (2012). Metal-organic framework materials as chemical sensors. Chem. Rev..

[44] Wei C.T., Li X., Xu F.G., Tan H.L., Li Z., Sun L.L., Song Y.H. (2014). Metal organic framework-derived anthill-like Cu@carbon nanocomposites for nonenzymatic glucose sensor. Anal. Methods.

[45] Xu H., Rao X., Gao J., Yu J., Wang Z., Dou Z., Cui Y., Yang Y., Chen B., Qian G. (2012). A luminescent nanoscale metal-organic framework with controllable morphologies for spore detection. Chem. Commun..

[46] Rozand C. (2014). Paper-based analytical devices for point-of-care infectious disease testing. Eur. J. Clin. Microbiol. Infect. Dis..

[47] Wu A., Gu Y., Beck C., Iqbal Z., Federici J.F. (2014). Reversible chromatic sensor fabricated by inkjet printing TCDA-ZnO on a paper substrate. Sens. Actuators B Chem..

[48] Jokerst J.C., Adkins J.A., Bisha B., Mentele M.M., Goodridge L.D., Henry C.S. A paper based analytical device for the colorimetric detection of food borne pathogens. Proceedings of the 15th International Conference on Miniaturized Systems for Chemistry and Life Sciences.

[49] Vaseashta A., Dimova-Malinovska D. (2005). Nanostructured and nanoscale devices, sensors and detectors. Sci. Tech. Adv. Mater..

[50] Kang X., Pang G., Chen Q., Liang X. (2013). Fabrication of *Bacillus cereus* electrochemical immunosensor based on double-layer gold nanoparticles and chitosan. Sens. Actuators. B Chem..

[51] Dou W., Tang W., Zhao G. (2013). A disposable electrochemical immunosensor arrays using 4-channel screen-printed carbon electrode for simultaneous detection of *Escherichia coli* O157:H7 and *Enterobacter sakazakii*. Electrochim. Acta.

[52] Chen J.-K., Zhou G.-Y., Chang C.-J., Cheng C.-C. (2014). Label-free detection of DNA hybridization using nanopillar arrays based optical biosensor. Sens. Actuators. B Chem..

[53] Wang Y., Ping J., Ye Z., Wu J., Ying Y. (2013). Impedimetric immunosensor based on gold nanoparticles modified graphene paper for label-free detection of *Escherichia coli* O157:H7. Biosens. Bioelectron..

[54] Zhang X., Dou W.C., Zhan X.J., Zhao G.Y. (2012). A novel immunosensor for *Enterobacter sakazakii* based on multiwalled carbon nanotube/ionic liquid/thionine modified electrode. Electrochim. Acta.

[55] Dong J., Zhao H., Xu M., Ma Q., Ai S. (2013). A label-free electrochemical impedance immunosensor based on AuNPs/PAMAM-MWCNT-Chi nanocomposite modified glassy carbon electrode for detection of *Salmonella* Typhimurium in milk. Food Chem..

[56] Salam F., Uludag Y., Tothill I.E. (2013). Real-time and sensitive detection of *Salmonella* Typhimurium using an automated quartz crystal microbalance (QCM) instrument with nanoparticles amplification. Talanta.

[57] Pérez-López B., Merkoçi A. (2011). Nanomaterials based biosensors for food analysis applications. Trends Food Sci. Technol..

[58] Gilmartin N., O’Kennedy R. (2012). Nanobiotechnologies for the detection and reduction of pathogens. Enzyme Microb. Technol..

[59] Chen S.-H., Wu V.C.H., Chuang Y.-C., Lin C.-S. (2008). Using oligonucleotide-functionalized Au nanoparticles to rapidly detect foodborne pathogens on a piezoelectric biosensor. J. Microbiol. Methods.

[60] Yang G.J., Huang J.L., Meng W.J., Shen M., Jiao X.A. (2009). A reusable capacitive immunosensor for detection of *Salmonella* spp. Based on grafted ethylene diamine and self-assembled gold nanoparticle monolayers. Anal. Chim. Acta.

[61] Biacore. https://www.biacore.com/lifesciences/index.html.

[62] Leonard P., Hearty S., Quinn J., O’Kennedy R. (2004). A generic approach for the detection of whole *Listeria monocytogenes* cells in contaminated samples using surface plasmon resonance. Biosens. Bioelectron..

[63] Biosensing Instruments Ltd.. www.biosensingusa.com.

[64] Taylor A.D., Ladd J., Yu Q., Chen S., Homola J., Jiang S. (2006). Quantitative and simultaneous detection of four foodborne bacterial pathogens with a multi-channel SPR sensor. Biosens. Bioelectron..

[65] Leung A., Shankar P.M., Mutharasan R. (2007). A review of fiber-optic biosensors. Sens. Actuators. B Chem..

[66] Narsaiah K., Jha S.N., Bhardwaj R., Sharma R., Kumar R. (2012). Optical biosensors for food quality and safety assurance—A review. J. Food Sci. Technol..

[67] Ohk S.H., Bhunia A.K. (2013). Multiplex fiber optic biosensor for detection of *Listeria monocytogenes*, *Escherichia coli* O157:H7 and *Salmonella* enterica from ready-to-eat meat samples. Food Microbiol..

[68] Adley C., Ryan M.P., Bhunia A.K., Kim M.S., Taitt C.R. (2014). Conductometric biosensor for high throughput screening of pathogens in food. High Throughput Screening for Food Safety Assessment: Biosensor Technologies, Hyperspectral Imaging and Practical Applications.

[69] Yang X., Zitova A., Kirsch J., Fergus J.W., Overfelt R.A., Simonian A.L. (2012). Portable and remote electrochemical sensing system for detection of tricresyl phosphate in gas phase. Sens. Actuators. B Chem..

[70] Zhai D., Liu B., Shi Y., Pan L., Wang Y., Li W., Zhang R., Yu G. (2013). Highly sensitive glucose sensor based on Pt nanoparticle/polyaniline hydrogel heterostructures. ACS Nano.

[71] Su X.-L., Li Y. (2005). A QCM immunosensor for *Salmonella* detection with simultaneous measurements of resonant frequency and motional resistance. Biosens. Bioelectron..

[72] Mecea V.M. (2006). Is quartz crystal microbalance really a mass sensor?. Sens. Actuators A Phys..

[73] BCC Research Surface Acoustic Wave (SAW) Devices: Technologies and Global Markets. http://www.bccresearch.com/report/download/report/ias039a.

[74] Rocha-Gaso M.I., March-Iborra C., Montoya-Baides A., Arnau-Vives A. (2009). Surface generated acoustic wave biosensors for the detection of pathogens: A review. Sensors.

[75] Reyes P.I., Li J., Duan Z., Yang X., Cai Y., Huang Q., Lu Y. (2013). ZnO surface acoustic wave sensors built on zein-coated flexible food packages. Sens. Lett..

[76] Sharma H., Mutharasan R. (2013). Review of biosensors for foodborne pathogens and toxins. Sens. Actuators. B Chem..

[77] Barthelmebs L., Calas-Blanchard C., Istamboulie G., Marty J.-L., Noguer T., Giardia M.T., Rea G., Berra B. (2010). Biosenosrs as analytical tools in food fermentation industry. Bio-Farms for Nutraceuticals; Functional Food and Safety Control by Biosensors.

[78] Ikeda T., Kato K., Maeda M., Tatsumi H., Kano K., Matsushita K. (1997). Electrocatalytic properties of *Acetobacter aceti* cells immobilized on electrodes for the quinone-mediated oxidation of ethanol. J. Electroanal. Chem..

[79] Lei Y., Mulchandani P., Chen W., Mulchandani A. (2004). Direct determination of *p*-nitrophenyl substituent organophosphorus nerve agents using a recombinant *Pseudomonas putida* JS444-modified clark oxygen electrode. J. Agric. Food Chem..

[80] Nakamura H., Suzuki K., Ishikuro H., Kinoshita S., Koizumi R., Okuma S., Gotoh M., Karube I. (2007). A new BOD estimation method employing a double-mediator system by ferricyanide and menadione using the eukaryote *Saccharomyces cerevisiae*. Talanta.

[81] Kara S., Keskinler B., Erhan E. (2009). A novel microbial BOD biosensor developed by the immobilization of *P. syringae* in micro-cellular polymers. J. Chem. Technol. Biotechnol..

[82] Su L., Jia W., Hou C., Lei Y. (2011). Microbial biosensors: A review. Biosens. Bioelectron..

[83] Paniel N., Baudart J., Hayat A., Barthelmebs L. (2013). Aptasensor and genosensor methods for detection of microbes in real world samples. Methods.

[84] Patterson A.S., Hsieh K., Soh H.T., Plaxco K.W. (2013). Electrochemical real-time nucleic acid amplification: Towards point-of-care quantification of pathogens. Trends Biotechnol..

[85] Pedrero M., Campuzano S., Pingarron J.M. (2009). Electroanalytical sensors and devices for multiplexed detection of foodborne pathogen microorganisms. Sensors.

[86] Sun H., Zhang Y., Fung Y. (2006). Flow analysis coupled with PQC/DNA biosensor for assay of *E. coli* based on detecting DNA products from PCR amplification. Biosens. Bioelectron..

[87] Rodriguez M.I., Alocilja E.C. (2005). Embedded DNA-polypyrrole biosensor for rapid detection of *Escherichia coli*. IEEE Sens. J..

[88] Wu V.C., Chen S.H., Lin C.S. (2007). Real-time detection of *Escherichia coli* O157:H7 sequences using a circulating-flow system of quartz crystal microbalance. Biosens. Bioelectron..

[89] Lermo A., Campoy S., Barbe J., Hernandez S., Alegret S., Pividori M.I. (2007). *In situ* DNA amplification with magnetic primers for the electrochemical detection of food pathogens. Biosens. Bioelectron..

[90] Zhu X., Zhou X., Xing D. (2012). Nano-magnetic primer based electrochemiluminescence-polymerase chain reaction (NMPE-PCR) assay. Biosens. Bioelectron..

[91] Pöhlmann C., Wang Y., Humenik M., Heidenreich B., Gareis M., Sprinzl M. (2009). Rapid, specific and sensitive electrochemical detection of foodborne bacteria. Biosens. Bioelectron..

[92] Mairhofer J., Roppert K., Ertl P. (2009). Microfluidic systems for pathogen sensing: A review. Sensors.

[93] Berdat D., Martin Rodriguez A.C., Herrera F., Gijs M.A. (2008). Label-free detection of DNA with interdigitated micro-electrodes in a fluidic cell. Lab Chip.

[94] Yeung S.W., Lee T.M., Cai H., Hsing I.M. (2006). A DNA biochip for on-the-spot multiplexed pathogen identification. Nucleic Acids Res..

[95] Fang T.H., Ramalingam N., Xian-Dui D., Ngin T.S., Xianting Z., Lai Kuan A.T., Peng Huat E.Y., Hai-Qing G. (2009). Real-time PCR microfluidic devices with concurrent electrochemical detection. Biosens. Bioelectron..

[96] Jha S.K., Chand R., Han D., Jang Y.C., Ra G.S., Kim J.S., Nahm B.H., Kim Y.S. (2012). An integrated PCR microfluidic chip incorporating aseptic electrochemical cell lysis and capillary electrophoresis amperometric DNA detection for rapid and quantitative genetic analysis. Lab Chip.

[97] Ahmed M.U., Nahar S., Safavieh M., Zourob M. (2013). Real-time electrochemical detection of pathogen DNA using electrostatic interaction of a redox probe. Analyst.

[98] Ellington A.D., Szostak J.W. (1990). *In vitro* selection of RNA molecules that bind specific ligands. Nature.

[99] Amaya-Gonzalez S., de-los-Santos-Alvarez N., Miranda-Ordieres A.J., Lobo-Castanon M.J. (2013). Aptamer-based analysis: A promising alternative for food safety control. Sensors.

[100] Zelada-Guillen G.A., Bhosale S.V., Riu J., Rius F.X. (2010). Real-time potentiometric detection of bacteria in complex samples. Anal. Chem..

[101] Ning Y., Li W., Duan Y., Yang M., Deng L. (2014). High specific DNAzyme-aptamer sensor for *Salmonella paratyphi* a using single-walled nanotubes-based dual fluorescence-spectrophotometric methods. J. Biomol. Screen..

[102] Jyoti A., Vajpayee P., Singh G., Patel C.B., Gupta K.C., Shanker R. (2011). Identification of environmental reservoirs of nontyphoidal salmonellosis: Aptamer-assisted bioconcentration and subsequent detection of *Salmonella* Typhimurium by quantitative polymerase chain reaction. Environ. Sci. Technol..

[103] Schmelcher M., Loessner M.J. (2014). Application of bacteriophages for detection of foodborne pathogens. Bacteriophage.

[104] Tawil N., Sacher E., Mandeville R., Meunier M. (2014). Bacteriophages: Biosensing tools for multi-drug resistant pathogens. Analyst.

[105] Nanosphere. www.nanosphere.us.

[106] 3M. http://www.3m.com/.

[107] Neogen. www.neogen.com.

[108] Serosep. www.serosep.com.

[109] Veredus Laboratories. www.vereduslabs.com.

[110] BIO phage PHARMA Inc.. http://www.biophagepharma.net/index.php/en/.

[111] Easy Life Science. http://www.elice.fr/.

[112] STRATOPHASE™. http://www.stratophase.com/.

[113] Available Real-Time PCR Cyclers. http://cyclers.gene-quantification.info/.

